# *Mycobacterium arosiense*, an unexpected cause of osteomyelitis in a patient with sarcoidosis: a case report

**DOI:** 10.1186/s12879-019-4638-3

**Published:** 2019-11-26

**Authors:** Didi Bang, Erik Michael Rasmussen, Aase Bengaard Andersen

**Affiliations:** 1 Virus & Microbiological Special Diagnostics, Infectious Disease Preparedness, Statens Serum Institut, Copenhagen, Denmark; 2 International Reference Laboratory of Mycobacteriology, Statens Serum Institut, Copenhagen, Denmark; 3 Department of infectious Diseases, Rigshospitalet, Copenhagen, Denmark

**Keywords:** Nontuberculous myobacteria, *Mycobacterium arosiense*, *Mycobacterium avium* complex, Vertebral osteomyelitis, Bone infection

## Abstract

**Background:**

Nontuberculous mycobacteria belonging to the *Mycobacterium avium* complex are recognized as opportunistic pathogens to humans. *Mycobacterium arosiense* is one of the novel members of the *Mycobacterium avium* complex. The organism has only rarely been reported in human clinical cases and may be routinely misidentified.

**Case presentation:**

An adult male with a history of a discus prolapse and sarcoidosis presented with high fever and a strong back pain with projection to the extremities. A Magnetic Resonance Imaging scan of columna revealed a tumor suspect process at thoracic vertebrae 11/12 with changes at the second lumbar vertebra, which was partly removed by laminectomy. Biopsy smears revealed acid-fast bacilli and turned out to be *Mycobacterium tuberculosis* complex PCR negative. The routine line probe assay INNO-LiPa v2 (INNOGENETICS NV, Gent), which differentiates 16 mycobacterial species indicated the presence of a not readily identifiable NTM species. Whereas, the GenoType Mycobacterium CM v2.0 (HAIN Lifescience GmbH) that routinely differentiates 14 clinically relevant mycobacteria revealed a *Mycobacterium intracellulare* species. However, additional diagnostic sequencing of the 16S rRNA gene confirmed the presence of a *Mycobacterium arosiense* species.

**Conclusions:**

This is the second unusual case of osteomyelitis with clinical significance ever to be reported, caused by *Mycobacterium arosiense* and complicated by an underlying sarcoidosis. *Mycobacterium arosiense* has rarely been reported clinically and the first description of the species was identified as the cause of osteomyelitis in a child with a hereditary partial interferon gamma deficiency. Symptoms attributed to sarcoidosis waned on *Mycobacterium arosiense* treatment and it is inconclusive whether the patient ever suffered from sarcoidosis. *Mycobacterium arosiense* was misidentified by the GenoType as *Mycobacterium intracellulare* and implicates that the diagnosis requires supplemental sequencing of the 16S rRNA gene.

## Background

Most nontuberculous mycobacteria (NTM) may occasionally be the causative agents of osteomyelitis. The *Mycobacterium avium* complex (MAC) bacteria are NTM recognized as opportunistic pathogens of humans. In Denmark, *Mycobacterium avium* complex has been isolated from nonpulmonary sites in 16% of cases [1]. Vertebral osteomyelitis is a rare manifestation of MAC in persons with human immunodeficiency virus or suffering from other immunocompromise, but is even less common in persons without immunocompromising conditions.

Classically, the MAC predominately consisted of two species, *Mycobacterium avium* and *Mycobacterium intracellulare*. However, new molecular methods of nucleotide identities reveal that the MAC currently consists of 12 valid species [2]. *Mycobacterium arosiense,* a slow growing, yellow-pigmented, scotochromogenic, NTM is one of the novel species of the MAC. *Mycobacterium arosiense* was isolated for the first time from osteomyelitic lesions of a child with an underlying partial interferon gamma (IFN-γ) receptor alpha-1 deficiency in 2008 [3]. The reporting of pathogens with clinical relevance within the MAC is of utmost importance. *Mycobacterium arosiense* may not readily be routinely identified. We report here a rare case of vertebral osteomyelitis in an adult with a presumed underlying diagnosis of sarcoidosis.

## Case presentation

An adult male of 35-years of age with a medical history of a discus prolapse more than a decade ago and a diagnosis of sarcoidosis 6 years previously based on a chest X-ray with bilateral infiltrations, and hilar lymphadenopathy. A testicular biopsy taken for fertility assessment, showed non-casseous changes. The biopsy was smear-negative for acid-fast bacilli (AFB) and cultures for bacteria and mycobacteria were negative. In support of the sarcoidosis diagnosis elevated C-reactive protein (CRP), erythrocyte sedimentation rate, and angiotensin-converting enzyme (ACE) levels were found. PET-CT scans showed metabolically active glands in the retroperitoneum and activity in the left upper lobe of the lungs. The sarcoidosis had been treated with steroids intermittently for years and was currently treated with prednisolone 2.5 mg q.d monotherapy at the time of diagnosis, and monitored with ACE measurements. Otherwise, the patient was healthy with no known allergies, other immune deficiencies, cardiovascular disorders or diabetes mellitus.

One week prior to ambulatory care the patient presented with uncharacteristic symptoms and experienced strong back pains with a stinging pain in the extremities and left side of the chest not unlike prior discus prolapse symptoms. However, with a high fever and sweat tendency. Mild analgesics in the form of oral *paracetamol* and *NSAID* caused symptoms to wane and the temperature normalized. A neurological examination was normal and no neurological focal signs or urinary functional problems were found. A slight dry cough and breathlessness, and a high pulse rate of 130 were observed. An elevated CRP of 70 (< 10 mg/L) and a normal ACE level of 93 (30–115 U/l) was interpreted as no sign of sarcoidosis reactivation. The p-basic phosphatase level was slightly elevated to 181 U/L (30–105), which however normalized. Electrocardiogram was normal. A chest X-ray showed regression of infiltrates but unchanged hilar lymphadenopathy. Sputum smear was negative for acid-fast bacilli and culture. A pulmonary function test showed reduced diffusion capacity and vital capacity to 70% of expected, which until now had been considered due to sarcoidosis.

One month later a Magnetic Resonance imaging scan of columna revealed a tumor suspect process extradurally at thoracic vertebrae 11/12 with changes at the second lumbar vertebra. A fortnight later, decompression of thoracic vertebrae and lumbar biopsies were performed. Thoracic laminectomy revealed a tumor-like process from which pus exuded. The tumor was partially removed and the biopsies and pus were sent for smear, bacterial culture including Mycobacteria and pathology. Smear revealed few to moderate AFB in all four biopsies. On suspicion of a diagnosis of tuberculosis anti-tuberculosis oral medication was started with rifampin 600 mg q.d., isoniazid 300 mg q.d., ethambutol 1200 mg q.d., pyrazinamide 2000 mg q.d., and pyridoxine 20 mg q.d. The prednisolone treatment was ceased. At this point the CRP was elevated at 59 (< 10 mg/L) with normal hematology, liver and kidney laboratory results. A human immunodeficiency virus test was negative. Sputum samples for tuberculosis diagnostics were negative. Biopsy material turned out to be *Mycobacterium tuberculosis* complex PCR negative, and the routine line probe assay INNO-LiPa v2 (INNOGENETICS NV, Gent), which differentiates 16 mycobacterial species indicated the presence of a not readily identifiable NTM species. On suspicion of tuberculosis, anti-tuberculosis treatment was continued. Additional identification by the line probe assay GenoType Mycobacterium CM v2.0 (HAIN Lifescience GmbH), which differentiates 14 clinically relevant mycobacteria indicated the presence of *a Mycobacterium intracellulare*.

Almost 3 weeks after the biopsy was performed supplemental diagnostic sequencing of the 16S rRNA gene revealed a rare *Mycobacterium arosiense*, a NTM belonging to the *Mycobacterium avium* complex [2]. Medication was changed to rifabutin 300 mg q.d., clarithromycin 500 mg b.i.d. and ethambutol 1200 mg q.d. Anti-tuberculosis treatment was ceased. However, signs of bone marrow toxicity in the form of leucopenia 0.7–0.9 × 10^9^/L, low CD4 cell counts at 150–200 × 10^6^ cells/L, and thrombocytopenia at 143 × 10^9^/L were observed. The rifabutin dose was decreased to 150 mg q.d.. Immunoglobulin subclasses results were unspecific. The patient completed 6 months oral anti-NTM medication before ceasing medication. Respiratory breathlessness that was present through many years disappeared during NTM treatment.

## Discussion and conclusions

*Mycobacterium arosiense* has rarely been reported clinically and the first description of the species was identified from several osteomylitic bone lesion sites of a girl with a hereditary partial IFN-γ receptor alpha-1 deficiency [3]. Complete absence of the IFN-γ receptor alpha-1 results in a deficiency that leads to early onset and severe mycobacterial infections [4]. Recessive null IFN-γ receptor-1 mutations preclude the expression of IFN-γ receptor-1 on the cell surface, or recognition of the ligand IFN-γ by surface expressed receptors. Affected patients therefore fail to respond to IFN-γ and have high concentrations of IFN-γ after infection. Numerous reports from such patients that have suffered from disseminated infections caused by NTM and/or Bacille Calmette-Guerin vaccines, with impaired granuloma formation have been described. Only one other clinical case of pulmonary disease has ever been reported, where *Mycobacterium arosiense* was found in an adult male with a history of Hodgkin’s lymphoma, gastric carcinoma and splenectomy [5].

We report the second unusual osteomyelitic case ever reported complicated by an underlying sarcoidosis. According to American Thoracic Society guidelines [6] several factors increase the likelihood of clinical significance of NTM isolates, including recovery from multiple specimens or sites, recovery of the organism in large quantities, AFB smear-positive specimens, or recovery of the organism from sterile sites such as bone material. The present case fulfills all these criteria of clinical significance.

Immune analysis showed the IFN-γ receptor signal ring was normal with no suspicion of changes in the interleukin-12/IFN-γ axis signal ring. Analysis of immunoglobulin (Ig) G classes and subclasses were normal for IgA, IgM and IgG, but were decreased for IgG2 and IgG4. Humoral B lymphocytes were slightly reduced. In reflection of slight leucopenia, with no thrombocytopenia, and normal hemoglobin throughout it was difficult to conclude on an actual immune defect. It is inconclusive whether the patient ever suffered from sarcoidosis. The previously observed increase of ACE may have been due to granuloma dissolving and not due to progression in sarcoidosis.

Sarcoidosis is an inflammatory condition of the immune system with development and accumulation of granulomas commonly involving several organs [7]. The cause of sarcoidosis is still unknown, although environmental, immunological and bacterial causes have been implicated as triggers of immune responses resulting in sarcoidosis. Scientists have recovered mycobacteria and propionibacteria RNA and DNA from sarcoidal tissues. Likewise, serum antibodies to mycobacterial antigens, including recombinant *Mycobacterium tuberculosis* katG, heat-shock protein 70 and 18, and mycolyl transferase antigen 85A.19 have been retrieved from patients with sarcoidosis.

Retrospectively, the patient experienced neuritis vestibularis and a facialis paresis together with pulmonary symptoms, which were perceived as sarcoidosis. The patient also had bilateral hilar lymph node swelling, which all could be explained by sarcoidosis, and the biopsy showed signs of sarcoidosis and not NTM. However, the fact that the sarcoidosis symptoms disappeared on treatment for a NTM, support the theory that the infection may have been present for a long time. Infiltrations on Chest-X rays resolved, although some degree of hilar lymphadenopathy remained. As proposed chronic corticosteroid therapy may pose a greater risk for MAC vertebral osteomyelitis than previously recognized [8].

Follow-up several months after the medication was ceased revealed that the patient continued to have leucopenia at 3 × 10^9^/L with low granulocytes at 1.4 × 10^9^/L and lymphocytes at 0.76 × 10^9^/L. The thrombocyte level had normalized and the presence of a permanent immunosuppression was discussed though not confirmed.

As in the present case *Mycobacterium arosiense* has been misidentified by the GenoType CM as *Mycobacterium intracellulare* [5]. Implications of misidentification indicate that *Mycobacterium arosiense* may be less rare than considered and supplemental diagnostic sequencing of the 16S rRNA gene on all routine *Mycobacterium intracellulare* strains is required. The consequences of differentiation of the *Mycobacterium arosiense* from the MAC are unknown. Some organisms within the MAC complex such as *Mycobacterium chimaer*a have poor treatment responses [9]. The recent introduction of affordable sequencing platforms may increase our knowledge on the management and treatment of patients suffering from *Mycobacterium arosiense* infections.

## Data Availability

All data generated or analyzed during this study are included in this published article.

## Abbreviations

ACE: Angiotensin-converting enzyme
AFB: Acid-fast bacilli
CRP: C-reactive protein
CT: Computed tomography
IFN-γ: Interferon gamma
Ig: Immunoglobulin
MAC: *Mycobacterium avium* complex
NTM: Nontuberculous mycobacteria
PET: Positron emission tomography
